# Structural basis of translation inhibition by cadazolid, a novel quinoxolidinone antibiotic

**DOI:** 10.1038/s41598-019-42155-4

**Published:** 2019-04-04

**Authors:** Alain Scaiola, Marc Leibundgut, Daniel Boehringer, Patrick Caspers, Daniel Bur, Hans H. Locher, Georg Rueedi, Daniel Ritz

**Affiliations:** 1 ETH Zurich, Department of Biology, Zurich, Switzerland; 2 Actelion Pharmaceuticals Ltd, Allschwil, Switzerland; 3 Present Address: Idorsia Pharmaceuticals Ltd, Allschwil, Switzerland; 4 Present Address: Polyphor Ltd, Allschwil, Switzerland

## Abstract

Oxazolidinones are synthetic antibiotics used for treatment of infections caused by Gram-positive bacteria. They target the bacterial protein synthesis machinery by binding to the peptidyl transferase centre (PTC) of the ribosome and interfering with the peptidyl transferase reaction. Cadazolid is the first member of quinoxolidinone antibiotics, which are characterized by combining the pharmacophores of oxazolidinones and fluoroquinolones, and it is evaluated for treatment of *Clostridium difficile* gastrointestinal infections that frequently occur in hospitalized patients. *In vitro* protein synthesis inhibition by cadazolid was shown in *Escherichia coli* and *Staphylococcus aureus*, including an isolate resistant against linezolid, the prototypical oxazolidinone antibiotic. To better understand the mechanism of inhibition, we determined a 3.0 Å cryo-electron microscopy structure of cadazolid bound to the *E. coli* ribosome in complex with mRNA and initiator tRNA. Here we show that cadazolid binds with its oxazolidinone moiety in a binding pocket in close vicinity of the PTC as observed previously for linezolid, and that it extends its unique fluoroquinolone moiety towards the A-site of the PTC. In this position, the drug inhibits protein synthesis by interfering with the binding of tRNA to the A-site, suggesting that its chemical features also can enable the inhibition of linezolid-resistant strains.

## Introduction

In all living cells, ribosomes are responsible for translating the genetic information from mRNAs to proteins. Due to this central role in protein synthesis ribosomes are frequently targeted by antibiotics that specifically inhibit bacterial translation by binding to the prokaryotic 70S but not eukaryotic 80S ribosomes^1,2^. Many of the clinically used antibiotic classes (chloramphenicols, macrolides, lincosamides, streptogramins and oxazolidinones) inhibit protein synthesis by binding to the peptidyl transferase centre (PTC), the catalytic centre of the ribosome, located in the large ribosomal subunit (50S subunit in prokaryotes), and to the nascent polypeptide exit tunnel of the 50S subunit^3^. The mechanism of how these antibiotics inhibit protein synthesis by interfering with the peptidyl transferase reaction or preventing the diffusion of the nascent polypeptide chain through the ribosomal tunnel are quite well understood based on biochemical, genetic and structural studies^3–6^.

Among the antibiotics that interfere with the peptidyl transferase reaction, oxazolidinones have been in the focus of antibacterial discovery research for many years, and two drugs have been approved for the treatment of infections due to susceptible Gram-positive pathogens^7,8^. Linezolid (LZD) was the first clinically used antibiotic of this class, but a wide variety of oxazolidinone analogues were designed in recent years to improve efficiency against resistant strains^9,10^.

**Figure 1:**
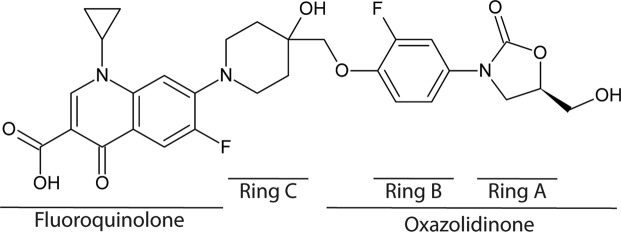
Chemical structure of cadazolid (CDZ) with the names of the different moieties indicated below.

Cadazolid (CDZ, Fig. 1) belongs to a new class of antibiotics termed quinoxolidinones and is in development for the treatment of *Clostridium difficile* infections (CDI). CDI is a major health care issue due to its morbidity and mortality, mainly for elderly hospitalized patients^11^, and due to the occurrence of hypervirulent drug-resistant strains^11,12^. The molecule combines elements of an oxazolidinone with a fluoroquinolone moiety, resulting in unique biological and physicochemical properties. CDZ showed potent *in vitro* activity against *C. difficile*^13^ and has an antibacterial spectrum largely limited to Gram-positive bacteria, while activity against Gram-negative bacteria is weak or not detectable^14^, consistent with the observed limited impact on bacteria of the normal gut microflora in an *in vitro* human gut model^15^. In phase 2 clinical trials in CDI, CDZ demonstrated clinical cure rates similar to vancomycin with lower recurrence rates^16^. Two phase 3 clinical trials have been completed recently (NCT01983683, NCT01987895)^17^. The propensity of spontaneous resistance development *in vitro* is low, and CDZ retained activity against quinolone-resistant as well as linezolid-resistant strains and did not select for strains with significantly increased minimum inhibitory concentrations (MIC) for fluoroquinolones or LZD, indicating an absence of cross resistance^14,18^. CDZ is therefore a particularly interesting new drug in the fight against multidrug-resistant pathogens.

In this study, we determined the cryo-electron microscopy (cryo-EM) structure of an *E. coli* 70S ribosome in complex with mRNA, a P-site tRNA and CDZ, resolved at 3.0 Å. We observe that CDZ binds to the same binding pocket of the 50S subunit as LZD in the vicinity of the PTC^4,19^. The structure reveals CDZ-specific differences in binding and explains the potency of CDZ against linezolid-resistant strains.

## Results

### Inhibition of protein synthesis

In previous studies, we established that CDZ leads to a reduced *de novo* protein synthesis and that translation can be inhibited *in vitro* in *C. difficile*^13^. To expand the understanding about the protein synthesis inhibition, cell-free coupled transcription/translation assays were performed with extracts prepared from *E. coli* and two *S. aureus* strains.

CDZ potently inhibited *in vitro* translation in cell-free extracts from *E. coli* and from *S. aureus* linezolid-susceptible and -resistant strains (IC_50_ 0.24–0.33 µM; Table 1, Supplementary Fig. S1). In contrast, LZD was significantly less potent (IC_50_ 2.6 µM) and its activity was further reduced when using an extract from the *S. aureus* linezolid-resistant strain (IC_50_ 7.03 µM). In *S. aureus*, IC_50_ values were mirrored by the minimum inhibitory concentrations (MICs), which suggests that inhibition of protein synthesis is the primary mode of action in these strains. Furthermore, in contrast to LZD, the activity of CDZ is hardly affected by the 23S rRNA mutation G2576U or the methylation by Cfr (Supplementary Table S1 and Fig. S2). Not surprisingly, in *E. coli* the LZD and CDZ potency against a wt-strain is very low or is not measurable, however inhibitory activity can be detected in a *tolC* mutant that lacks the outer membrane drug efflux protein TolC (Table 1). Therefore, to characterize the binding of CDZ to the bacterial ribosome, compare its binding mode to the one of LZD and to determine the role of the quinolone moiety, we elucidated the structure of the *E. coli* 70S ribosome in complex with CDZ.

**Table 1:** *In vitro* transcription/translation assays for cadazolid and linezolid against *E. coli* wt ATCC 25922*; S. aureus* wt ATCC 29213*; S. aureus* S1 (LZD^R^). The minimal inhibitory concentrations are also shown.

		Cadazolid	Linezolid
*E. coli* ATCC 25922	Average (range)^a^	0.24 (0.13–0.34)	4.03 (1.92–5.39)
	MIC^b^	8/< = 0.063^c^	>32/8^d^
*S. aureus* ATCC 29213	Average (range)	0.32 (0.31–0.35)	2.61 (1.12–4.15)
	MIC	0.5	2
*S. aureus* S1 LZD^R d^	Average (range)	0.33 (0.25–0.44)	7.03 (6.16–9.16)
	MIC^c^	1	32

^a^IC_50_ in µM (average value and range of three independent experiments).

^b^MIC in µg/ml (median value of at least 3 determinations).

^c^Measured against *E. coli* MG1655 *tolC::kan*.

^d^Homozygous G2576U in 23S rRNA.

### Cryo-EM structure of CDZ in complex with the bacterial ribosome

**Figure 2:**
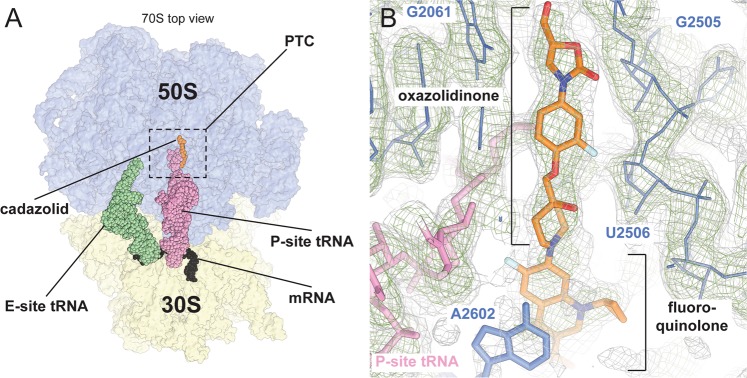
Overview of the *E. coli* 70S with bound tRNAs, mRNA and CDZ. (**a**) For orientation, the docked models of the 30S and 50S subunits are shown in yellow and blue, respectively. The mRNA and the P- and E-site tRNAs are shown as spheres in black, magenta and green. (**b**) Details of cadazolid and its surrounding area in the experimental 3.0 Å cryo-EM map. Two contour levels are shown in green and grey mesh.

*E. coli* 70S ribosomes incubated with CDZ (Fig. 1), mRNA and Phe-tRNA(Phe) and tRNA(fMet)_i_ were analysed by single particle cryo-EM. During the analysis, two dominant subpopulations were found in the sample corresponding to the 70S in complex with either two (P- and E-site) or three (A-, P-, and E-site) tRNAs (Supplementary Fig. S3). Both particle subsets were refined independently. The subset containing two tRNAs in P and E-sites of the ribosome revealed the presence of the quinoxolidinone antibiotic and was refined to higher resolution by focusing refinement on the 50S subunit, resulting in reconstructions reaching overall resolutions of 3.0 Å (Fig. 2, Supplementary Table S2 and Figs S3 and S4). The maps were interpreted by docking and refining an *E. coli* 50S subunit extracted from a crystal structure of the 70S ribosome (PDB: 4YBB^20^).

In the focused reconstruction we could unambiguously identify the density of the CCA-acyl end of a charged P-site tRNA and CDZ bound in the vicinity of the PTC (Fig. 2A,B and Supplementary Fig S4A). To verify the identity of the P-site tRNA in the decoding centre of the 30S subunit, we additionally performed a refinement focused on the small subunit, which resulted in a map resolved to 3.2 Å. Inspection of the anticodon stem loop and the codon-anticodon bases confirmed the identity of the P-site tRNA as a tRNA(fMet)_i_ at an AUG start codon (Supplementary Fig. S5). We observed density for the amino acid attached to the 3′ end of the tRNA bound to the P-site. Since it is likely that only a fraction of tRNA(fMet)_i_ purified from *E. coli* (tRNA Probes, LLC) was aminoacylated, the presence of the amino acid attached to the tRNA suggests that charged tRNAs preferentially bound to the 70S ribosomes (Supplementary Fig. S4A).

### Binding of cadazolid to the PTC

CDZ is composed of a system with five rings of which the A- and B-rings are found in all oxazolidinones, while the C-ring links this pharmacophore to the fluoroquinolone moiety (Fig. 1). CDZ is bound deeply in a pocket within the PTC. The quality of the density allowed unambiguous modelling of this part of the compound as well as the PTC area in the vicinity. Less-well defined density for the fluoroquinolone moiety of CDZ was also observed at lower contour levels, indicating intrinsic flexibility. The fluoroquinolone moiety is oriented towards the empty A-site tRNA binding pocket of the PTC, where it could be reasonably modelled in two alternative conformations: one with the cyclopropyl group pointing towards the P-site tRNA and one pointing towards residue A2507 of the rRNA (Fig. 2B and Supplementary Fig S4B).

**Figure 3:**
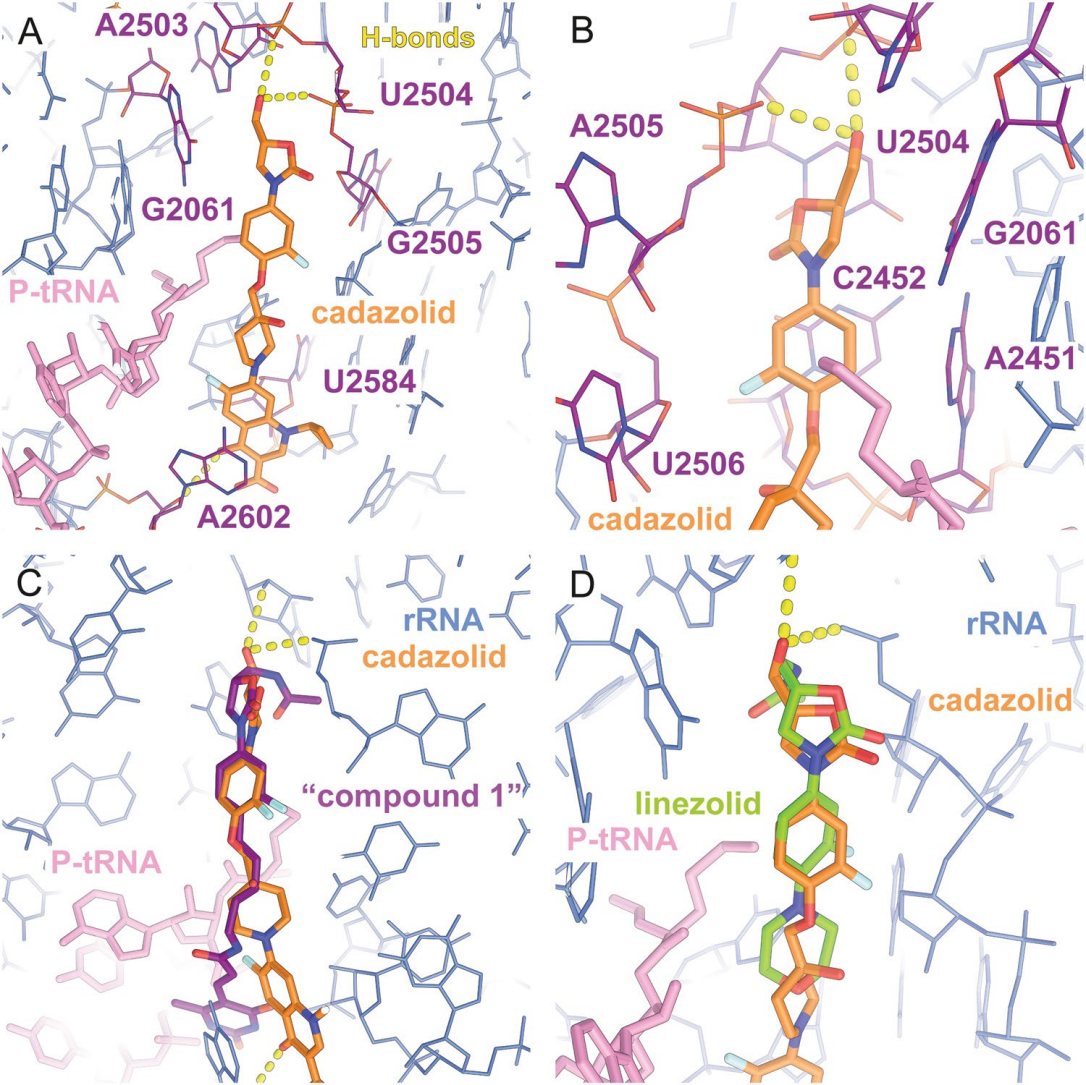
CDZ bound to the PTC pocket and superposition with structures of two other antibiotics of the oxazolidinone family. (**a**) Detailed view of CDZ and its interactions with the ribosome. CDZ is shown in orange, the P-site tRNA in pink and the rRNA in blue. The residues forming the pocket where CDZ binds are highlighted in purple. CDZ is within hydrogen-bonding distance of the backbones of A2503 and U2504 (yellow dashes). (**b**) Close-up view of the A- and B-rings of CDZ. The B-ring is stacking onto residue C2452 while being sandwiched between residues U2506 and A2451. (**c**) Overlay of CDZ with “compound 1”^10,21^ or (**d**) LZD^19^. The B rings of both oxazolidinones coincide with ring B of CDZ, but the compounds differ in the ring A and C substitutions. “Compound 1”, the tail of which also interacts with A2602, is a hybrid of an oxazolidinone antibiotic and sparsomycin.

Overall, the oxazolidinone moiety of CDZ binds to the same pocket and in a similar orientation as other oxazolidinone antibiotics, with the A- and B-rings forming the main interface next to the polypeptide exit tunnel at the PTC (Fig. 3A–D)^4,19,21,22^. The fluorophenyl B-ring is stacking onto residue C2452 while being sandwiched between residues U2506 and A2451 (Fig. 3B). The position of the B-ring is similar to the one observed in a structure of a *Haloarcula marismortui* (*Hm*) 50S-LZD complex^19^ and the position predicted based on crosslinking studies and molecular modelling simulations using LZD with an *E. coli* model^23^. The 2-oxazolidone A-ring is bound to the PTC pocket in a similar fashion as oxazolidinones, in the close vicinity of G2061, stacking against U2504 with its hydroxymethyl tail oriented towards A2503 (Fig. 3A). However, this hydroxymethyl tail, which is significantly shorter than the acetamide tail found in LZD (Fig. 3D), is positioned within hydrogen-bonding distance to the backbone of either A2503 (3.5 Å) or G2505 (2.9 Å) (Fig. 3A).

### Mechanism of translation inhibition by cadazolid

**Figure 4:**
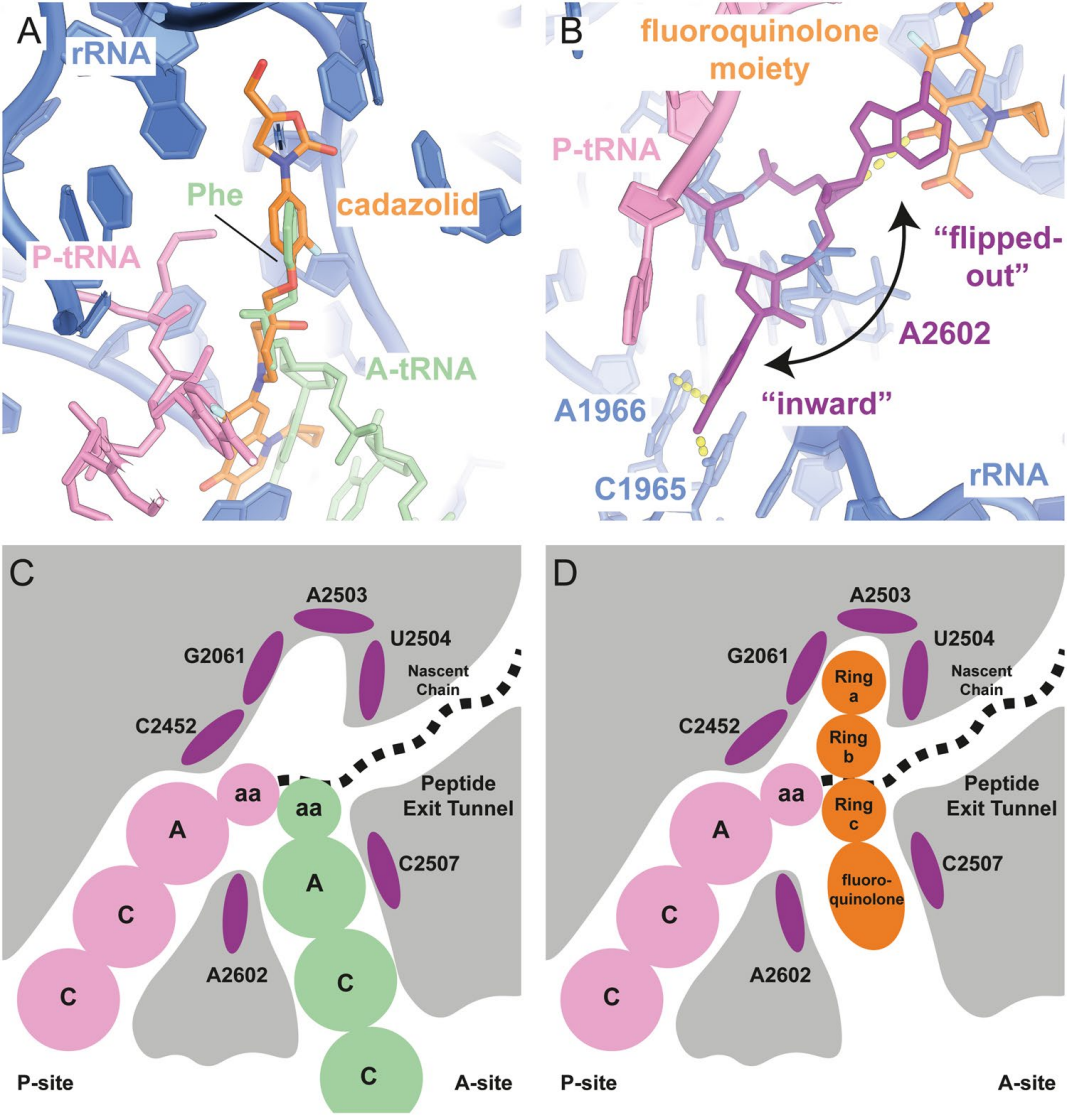
Observed mechanism of translation inhibition by cadazolid. (**a**) Overlay of an A-site tRNA (PDB 1VY4^51^) (light green) and CDZ (orange). Ring B and the fluoroquinolone moiety clash with an aminoacylated A-site tRNA, thereby preventing binding. (**b**) The two observed conformations of A2602. The fluoroquinolone moiety stacks with the “flipped-out” conformation to stabilize the PTC in a post-translocation conformation. (**c**,**d**) Schematic representation of the PTC with an A-site tRNA (**c**) or CDZ (**d**).

The specific mechanism of inhibition of translation by CDZ can be explained by the positions of the B-ring and the orientation of the fluoroquinolone moiety, which, together, would clash with a 3′ terminal nucleotide of an aminoacylated A-site tRNA (Fig. 4A) and hence prevent the elongation of the polypeptide chain by steric hindrance (Fig. 4C,D).

The fluoroquinolone moiety interacts with the backbone of U2584 and with one of the two alternative conformations observed for A2602 (Fig. 4B and Supplementary Fig. S4C). In the “inward” conformation, A2602 forms a non-standard base pair with C1965 and is positioned too far away from the antibiotic to become engaged in an interaction. However, in the other conformation, A2602 is “flipped-out”, thereby stacking on top of the fluoroquinolone moiety. A related stacking interaction had also been found in a complex with sparsomycin bound to the PTC^24^ as well as in a complex between the *Hm* 50S and “compound 1”, where the stacking partners are oriented at a different angle^21^ (Fig. 3C). Moreover, this highly conserved but flexible adenosine residue has been shown in a wide range of conformations, depending on the state of the ribosomes^25–27^, and was shown to be crucial for the peptidyl transferase reaction^24,28,29^. Interestingly, it was proposed that this base was linked to translocation of the tRNA^30^. By stacking with the base, the fluoroquinolone moiety likely stabilizes the P-site tRNA in a post-translocation state conformation resulting in a rigid and stably inhibited complex.

Compared to LZD, the acetamidomethyl tail on the A-ring is replaced by a hydroxymethyl group in CDZ, which is able to reach the bottom of the pocket to form a hydrogen bond with either A2503 or G2505. Together with the additional contacts between A2602 and the fluoroquinolone moiety, this could contribute to the overall higher potency of CDZ compared to LZD^13,18^.

Previous docking experiments revealed that changing the acetamide substituent at the C-5 position of the oxazolidinone ring in linezolid to a smaller hydroxymethyl substituent in tedizolid, a related oxazolidinone antibiotic, allowed the compound to bind ribosomes that either contained single nucleotide changes in the domain V of the 23S rRNA or modifications of A2503 through methylation by Cfr^31–34^. Interestingly, the smaller hydroxymethyl substituent on the oxazolidinone ring of CDZ would still allow binding of CDZ to ribosomes when A2503 is methylated, in agreement with the mode of binding observed in our cryo-EM structure (Supplementary Fig. S2).

Additionally, mutations in ribosomal protein uL3, which is located close to the CDZ binding pocket, were reported to be responsible for LZD resistance in *S. aureus*^32,35^ and were structurally linked to differences in the G2505 conformation^36^, resulting in a tighter binding pocket that would lead to a clash with LZD. A related mutation was found in *C. difficile* linezolid-resistant but CDZ-sensitive strains^18^. This mutation likely also causes reduction of the oxazolidinone binding pocket, showing that CDZ can still bind to a constricted pocket, most likely due to its shorter hydroxymethyl tail.

In the same study, a lysine-to-asparagine mutation in ribosomal protein uL4 was found in *C. difficile* with increased MIC and IC_50_ for CDZ. Despite being located 15 Å away from the bound CDZ, this residue is only separated from the antibiotic by one nucleotide, G2061, whose base lines the side of the pocket opposite to G2505. Interestingly, these mutants also have an increased MIC for LZD, indicating that this face of the pocket interacts with both LZD and CDZ in a similar fashion.

## Discussion

Cadazolid belongs to the new quinoxolidinone class of antibiotics that has been shown to inhibit both DNA and protein synthesis *in vitro* and in cellular assays. In *C. difficile*, CDZ predominantly acts by inhibition of protein synthesis, via its oxazolidinone pharmacophore which is also present in LZD, rather than by inhibition of DNA gyrase and topoisomerase IV^14^. We expanded our analyses and showed that CDZ is also potently inhibiting protein translation *in vitro* in extracts from *E. coli* and *S. aureus*. Mechanistic and structural studies suggested that oxazolidinones interfere with binding of the substrate tRNAs to the A-site and that tRNA bound to the P-site increases the affinity of binding of LZD to the ribosome^19,34^. These observations were further supported by the identification of mutations in the rRNA that confer resistance to LZD^31–34^. Indeed, extracts from *S. aureus* S1 that contain the G2576U mutation in the 23S rRNA gene were less susceptible to the inhibition by LZD. Still, we observed a large MIC shift suggesting that other mutations contributed to the resistance phenotype. (summarized in^32^).

In contrast, the activity of CDZ was not affected by any of those mutations that led to resistance to LZD. The activity against a panel of clinical *S. aureus* isolates either containing a mutation in the ribosomal RNA gene harbouring a methylase that is able to modify the 23S ribosomal RNA (*cfr*) was not reduced compared to wild-type isolates. Previously, only a single mutation in ribosomal protein uL4, which is located in proximity to the CDZ binding pocket, occurred in *C. difficile* during *in vitro* selection with CDZ^18^.

Our reconstruction of the *E. coli* 70S in complex with mRNA, E- and P-site tRNAs and CDZ reveals important mechanistic differences in the binding mode of CDZ, although the antibiotic is related to other compounds of the oxazolidinone family (Fig. 3C)^4,19^. The high potency of CDZ can now be explained structurally by the tight fitting of the rather rigid oxazolidinone moiety into the bottom of the pocket close to the PTC together with the role of CDZ in stabilization of the PTC in a P-site tRNA occupied/post-translocation state. Additionally, the binding mode of CDZ to the ribosomes observed in our structure rationalizes the activity against LZD-resistant bacterial isolates.

## Materials and Methods

### Bacterial strains and antimicrobial agents

Strains were obtained from ATCC (25922, 29213), Public Health England (formerly Health Protection Agency, Centre for Infections), London, GB (*S. aureus* S1), Merck (formerly Novagen) (*E. coli* BL21(DE3)) or are from the Actelion strain collection. Cadazolid (CDZ, ACT-179811) was synthesized at Actelion Pharmaceuticals Ltd. Linezolid (LZD) was obtained from commercial sources (AK Scientific, LZD 70412).

### Determination of the MIC

The MICs were determined using the micro dilution method recommended by the Clinical and Laboratory Standards Institute (CLSI).

### *In vitro* transcription/translation (IVTT) assays

The cell-free protein synthesis reactions were carried out based on a procedure published for *E. coli*^37^ and adapted for *S. aureus* as described previously for *C. difficile*^13^. To improve expression from extracts derived from *S. aureus*, the firefly luciferase reporter gene was resynthesized with codons optimized for *S. aureus* at GeneArt AG (Regensburg, Germany) and cloned into plasmid pSP-luc_NF Fusion (Promega), replacing the existing luciferase gene. In addition, a 180-bp-long *Bgl*II-*Hind*III promoter fragment of gene *abrB310* derived from *Clostridium acetobutylicum* was synthesized and cloned upstream of the codon-optimized luciferase gene for efficient translation, leading to plasmid pSPAbrlac-luc_Saur. *S. aureus* bacterial extracts were prepared according to a simplified *E. coli* procedure using a FastPrep®-24 instrument from Lucerna Chem AG (Cat No. 6004-500) and lysing matrix B (Lucerna Chem, Cat No. 116911100; silica spheres < 100 µm).

Test compounds diluted in 8% DMSO were added to reaction buffer containing bacterial extract and SP6 RNA polymerase, yielding final concentrations of 100, 20, 4, 0.8, 0.16, 0.04, 0.008, 0.0016, and 0 µM. Samples were mixed and pre-incubated for 10 min at RT before 100 ng plasmid pBestluc (*E. coli*) or 500 ng plasmid pSPAbrlac-luc_Saur (*S. aureus*) was added. After mixing, the plate was incubated at RT for 90 min. The luminescence was immediately measured after addition of the luciferase substrate Bright Glo (Promega, E2610) using a Tecan SpectralFluor Plus Reader (GeniosPro) instrument (parameters: integration time 1000 ms; intensity normal). Fifty percent inhibitory concentrations (IC50s) were calculated from the data.

### Ribosome purification

The ribosomes were purified from *E. coli* BL21(DE3) strain as described previously^38^ using associative conditions for the final gradient (20 mM HEPES-NaOH pH 7.4, 6.5 mM MgCl_2_, 0.5 mM EDTA, 60 mM NH_4_Cl, 5 mM DTT, 10–40% (w/v) sucrose).

### Complex formation

Due to low aqueous solubility of CDZ, the drug was dissolved in 100% DMSO resulting in a 7.85 mM solution, which was then diluted with the final buffer (20 mM HEPES-KOH pH 7.4, 10 mM MgCl_2_, 0.5 mM EDTA, 60 mM NH_4_Cl) to achieve a CDZ concentration of 1 mM. To assure proper binding, the 70S ribosomes (1.8 µM final concentration) were incubated overnight at 4 °C with an excess of CDZ (final concentration 10 µM) to allow the fairly large drug to have time to access the PTC and reach its binding equilibrium.

A 70S ribosome in complex with a P-site tRNA was then generated as described previously^39^. The mRNA was chemically synthetized (Dharmacon) with the sequence 5′-GCAAGGAGGUAAAAAUGUUUAAA-3′^39^. Incubated 70S ribosomes were first diluted to 180 nM while keeping the CDZ concentration constant. The mRNA (0.5 µM final concentration) was added to the 70S ribosome, and the mixture was incubated for 5 min at 37 °C. Next, the initiator tRNA(Met)_i_ (2 µM final concentration) (tRNA Probes, LLC) was added and the mixture was incubated for 30 min at 37 °C, followed by the addition of Phe-tRNA(Phe) (2 µM final concentration) (tRNA Probes, LLC) and another 30 min incubation at 37 °C.

### EM sample preparation and data collection

The grids were prepared using a FEI Vitrobot Mk4 (FEI Company), whose chamber was set to 100% humidity and 4 °C. 5 µL of the mixture prepared above was added to Quantifoil R2/2 holey carbon grids, which were previously coated with an additional thin layer of carbon and glow-discharged for 40 seconds with a current of 25 mA (negative). After 30 seconds incubation in the chamber, the excess liquid was blotted away for 11–24 seconds.

### EM data processing and reconstruction

One grid was used for data collection with a FEI Titan Krios cryo-transmission electron microscope (FEI company) operating at 300 KeV and equipped with a Falcon II direct electron detector (FEI company). For every hole of the holey carbon, four movies were collected at a magnification of 130’000x resulting in a pixel size of 1.079 Å/pixel, and the total dose was set to 40 electrons/Å^2^, resulting in 8328 movies.

The resulting movie frames were aligned using a patch-based algorithm (25 patches) and dose weighted using MotionCor2^40^. The contrast transfer function (CTF) parameters of the aligned micrographs were estimated using GCTF^41^, and the good micrographs were selected according to their CTF and micrograph quality. The particles of the resulting micrographs were picked using BATCHBOXER from the EMAN 1.9 package^42^. The ~773500 particles were then extracted and processed using Relion 2.0^43^. After initial 2D classification to remove the bad particles and the dissociated 50S ribosomal subunits, the 70S particles were refined according to the “gold standard”^44^ (Supplementary Fig. S3). These ~609000 aligned particles were then used for initial 3D classification to remove free 50S ribosomal subunits, immediately followed by a 3D classification with a mask around the intersubunit space to categorize for tRNA occupancy.

In this last classification, two main sets were visible: a first ribosomal complex containing tRNAs in its P- and E-sites, and a second complex containing tRNAs in its A-, P-, and E-sites. An additional classification was performed on the dataset containing two tRNAs to separate the different ratchet states of the ribosome, and the most populated one was selected. The two sets of particles were then refined independently reaching an overall resolution of 3.1 Å for the subpopulation with the two tRNAs and 2.9 Å for the subpopulation containing three tRNAs. The set containing two tRNAs was then subjected to a per-particle CTF estimation (GCTF^41^) followed by a refinement with a mask around the 50S subunit, post-processed and sharpened resulting in an overall resolution of 3.0 Å (Supplementary Fig. S6). Lastly, the same subset was refined with a mask around the 30S, reaching an overall resolution of 3.2 Å.

### Modelling, refinement and validation of the 50S large ribosomal subunit

First, an atomic model of *E. coli* 50S ribosomal subunit from the crystal structure of the 70S ribosome (PDB: 4YBB^20^) was docked into the EM density of the large subunit using UCSF Chimera^45^. The model was refined in reciprocal space using phenix.refine^46^ into the map as described previously^47^. After refinement, the differences between the EM map and the model were corrected by inspection of the F_obs_-F_calc_ difference Fourier maps using the programs O^48^ and COOT^49^. After rebuilding of the 50S subunit, inspection of the remaining difference density revealed the presence of an isolated CDZ molecule and a P-site tRNA in the PTC (Supplementary Fig. S4).

To model the CDZ molecule, coordinates were obtained in SDF format from the PubChem server (http://pubchem.ncbi.nlm.nih.gov) and, after conversion to PDB, were fed into PRODRG^50^ to obtain a topology file suitable for real and reciprocal space refinement. The CDZ model was fitted into the density using the real space fitting algorithm in COOT. The CCA-3′ termini of the tRNAs were built by docking and adjusting the corresponding model from a *Thermus thermophilus* 70S crystal structure (PDBs: 4QCM and 4QCN^51^).

The completed model was then refined to convergence by real space refinement using phenix.real_space_refine^52^. Validation of the atomic coordinates resulted in an optimal weighting of the model geometry versus the structure factors at a weight value of 1.4 (Supplementary Table S1). The estimated resolution of the model vs. map Fourier shell correlation (FSC) according to the FSC = 0.5 criterion coincided well with the resolution of the map established from the Fourier half set FSC correlation at the gold-standard criterion of 0.143 (Supplementary Fig. S6).

### Figure generation

Figures showing cryo-EM maps and the final model were generated using UCSF Chimera^45^ and PyMOL (The PyMOL Molecular Graphics System Version 1.8.4.0 Schrodinger).

## Supplementary information

Supplementary Figures and Table

## Data Availability

The final model of the 50S ribosomal subunit containing the CDZ molecule as well as the CCA-fMet of the P-site tRNA is available from the Protein Data Base (PDB) under accession number 6QUL. The cryo-EM map of whole 70S ribosome, as well as the reconstruction from the focused refinement around the 50S and 30S ribosomal subunits are deposited in the Electron Microscopy Data Base (EMDB) under the accession number 4639, 4638 and 4641, respectively.
