# Supplementary material for: Structural basis of translation inhibition by cadazolid, a novel quinoxolidinone antibiotic
Source: Sci Rep. 2019 Apr 4;9:5634. doi: 10.1038/s41598-019-42155-4 (PMC6449356; doi:10.1038/s41598-019-42155-4)
Supplement: Supplementary file 1 — Supplementary Figures and Table [file 41598_2019_42155_MOESM1_ESM.pdf]

Supplementary Tables and Figures:

Structural basis of translation inhibition by cadazolid, a novel quinoxolidinone antibiotic

Alain Scaiola[1], Marc Leibundgut[1], Daniel Boehringer[1], Patrick Caspers[2,3], Daniel Bur[2], Hans H. Locher[2,4], Georg Rueedi[2,3], Daniel Ritz[2,3] *

[1] ETH Zurich, Department of Biology
[2] Actelion Pharmaceuticals Ltd, Allschwil, Switzerland
[3] Present address: Idorsia Pharmaceuticals Ltd, Allschwil, Switzerland
[4] Present address: Polyphor Ltd, Allschwil, Switzerland

*Correspondence to daniel.ritz@idorsia.com

Supplementary Table S1: Activity of cadazolid and other antimicrobial drugs when tested against wildtype *S. aureus* strains and strains possessing molecularly characterized linezolid-resistance mechanisms using broth microdilution methods (CLSI).

	Cadazolid		Linezolid		Vancomycin	
S. aureus strain	MIC_{50}	MIC_{90}	MIC_{50}	MIC_{90}	MIC_{50}	MIC_{90}
wild-type (12*)	0.25	0.25	1	1	1	1
linezolid-nonsusceptible (26**)						
carrying *cfr* (12)	0.25	0.5	8	8	0.5	1
G2576U mutants (14)	0.5	0.5	8	16	1	1

* The 12 tested wild-type control strains include methicillin [oxacillin]-resistant [MRSA] strains [41.7%] from JMI Laboratories (North Liberty, Iowa).

** The 26 clinical strains tested included 12 cfr-positive and 14 23S rRNA mutants [G2576U].

Supplementary Table S2: EM Data collection, reconstruction and model statistics

**Creme Data collection**		
Microscope model	FEI Titan Krios cryo transmission electron microscope	
Detector model	FEI Falcon 2 Direct Electron Detector	
Number of micrographs collected	3035	
Pixel size (Å)	1.079	
Defocus range (µm)	0.5-3.0	
Voltage (kV)	300	
Electron dose (e/Å)	40	
	50S	30S
EMDB entry	4638	4641
PDB entry	6QUL	
Final number of particles	32’500	32’500
Resolution (masked, FSC=0.143) (Å)	2.97	3.24
Map sharpening B factor (Å^2)	-71.3	-80.9
**Refinement and model validation**		
Unit cell	P1	
a,b,c (Å)	210.41, 199.62,219.04	
α = β = γ (°)	90	
Resolution range (Å)	50.0-2.97	
Model resolution (model vs masked map, FSC=0.5)(Å)	3.05	
CC (masked)	0.85	
Molprobity clashscore	6.45	
EMRinger score	3.40	
Molprobity score	1.40	
**Protein**		
RMSD (bonds)	0.002	
RMSD (angles)	0.389	
Good rotamers (%)	98.70	
Ramachandran plot (%)		
Favoured	97.79	
Allowed	2.21	
Outliers	0	
**RNA**		
Correct sugar puckers (%)	99.73	
Good backbone conformation (%)	88.42	
Bad angles (%)	0.0	
Bad bonds (%)	0.0	

A

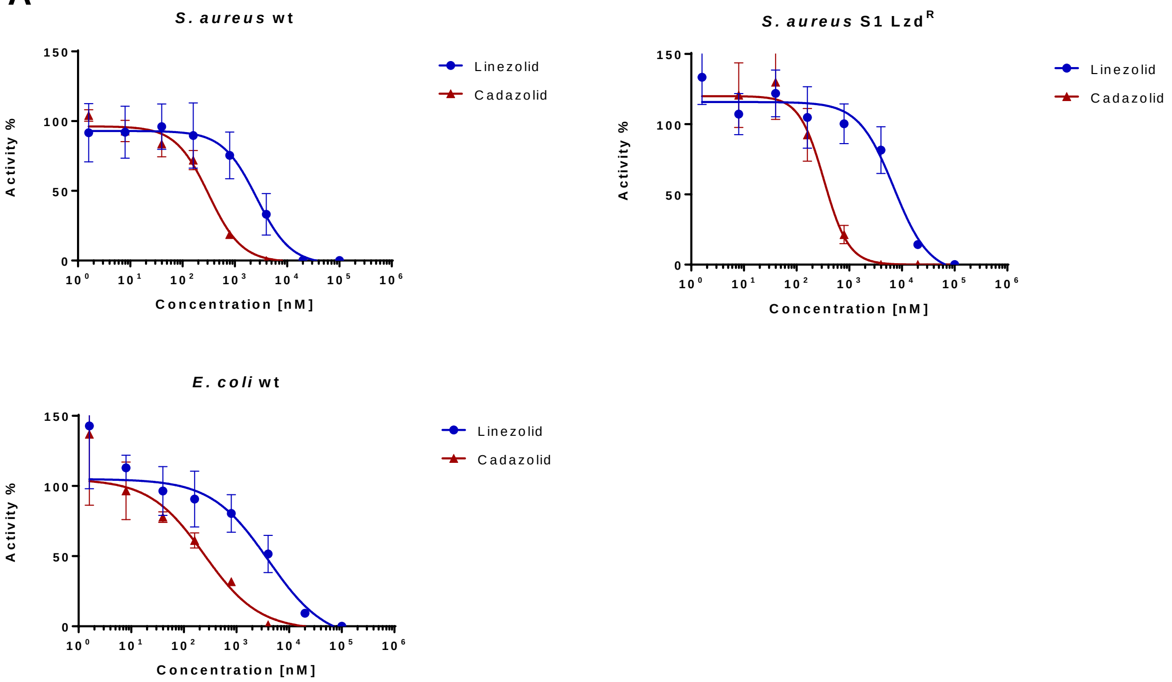

B

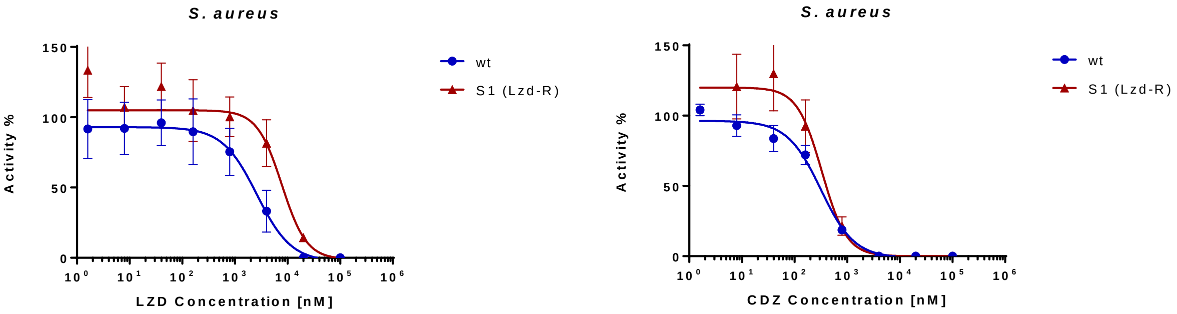

Supplementary Figure S1: *In vitro* transcription/translation dose-response curves for cadazolid and linezolid against *E. coli* wt ATCC 25922; *S. aureus* wt ATCC 29213; *S. aureus* S1 (LZD^R).

(a) Comparison of LZD and CDZ inhibitory response curves with extracts from *S. aureus* wt and LZD^R strains as well as *E. coli* wt.

(b) Comparison of LZD or CDZ dose-response curves for wild-type and LZD^R *S.aureus*.

Luminescence was measured in duplicate samples in three independent experiments. Activity was normalised to 100% for the no-drug controls of each experiment, plotted using GraphPad Prizm 7.04 as mean value with standard deviation and curve fitting done with nonlinear regression (log(inh) vs. response -- Variable slope, upper limit <105).

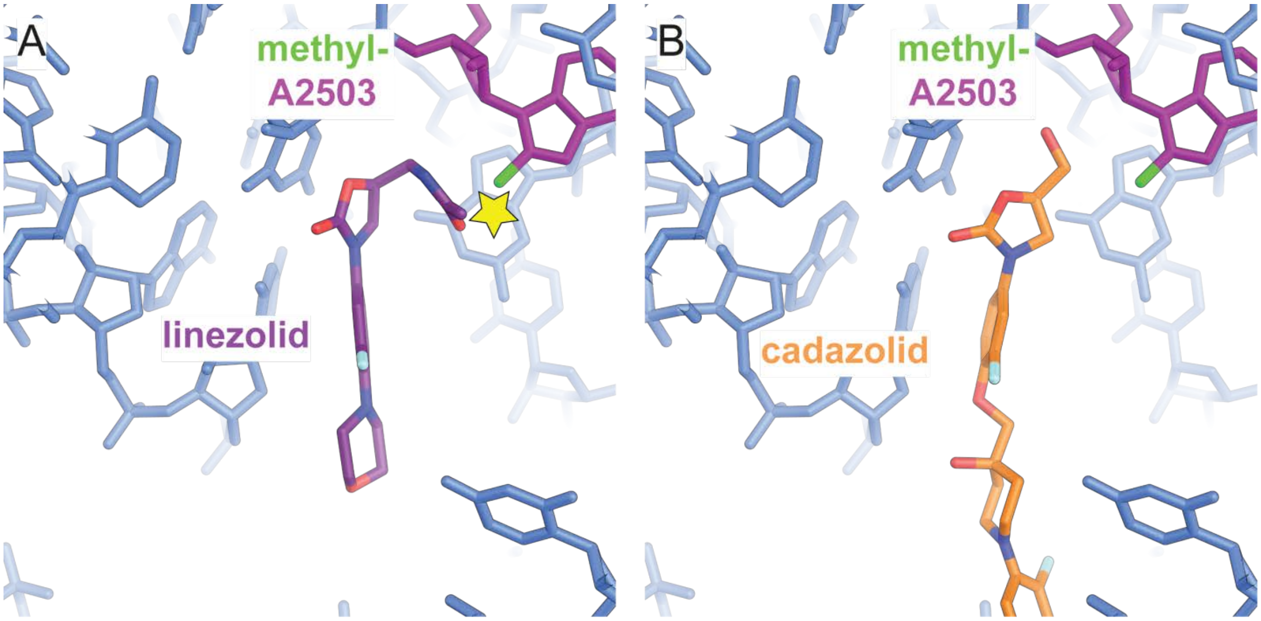

Supplementary Figure S2: Modelling of the PTC for the linezolid-resistant and cadazolid-sensitive *cfr* mutant

(a) Model of the PTC with a methylated nucleotide A2503, as it would occur in strains carrying the Cfr mutation. The extra methyl group would clash (highlighted by a star) with the acetamide moiety at the A ring of linezolid.

(b) Cadazolid would be less affected because the hydroxymethyl group of the A ring points into a different direction. This is in accordance with the obtained MIC results (Supplementary Table 1)

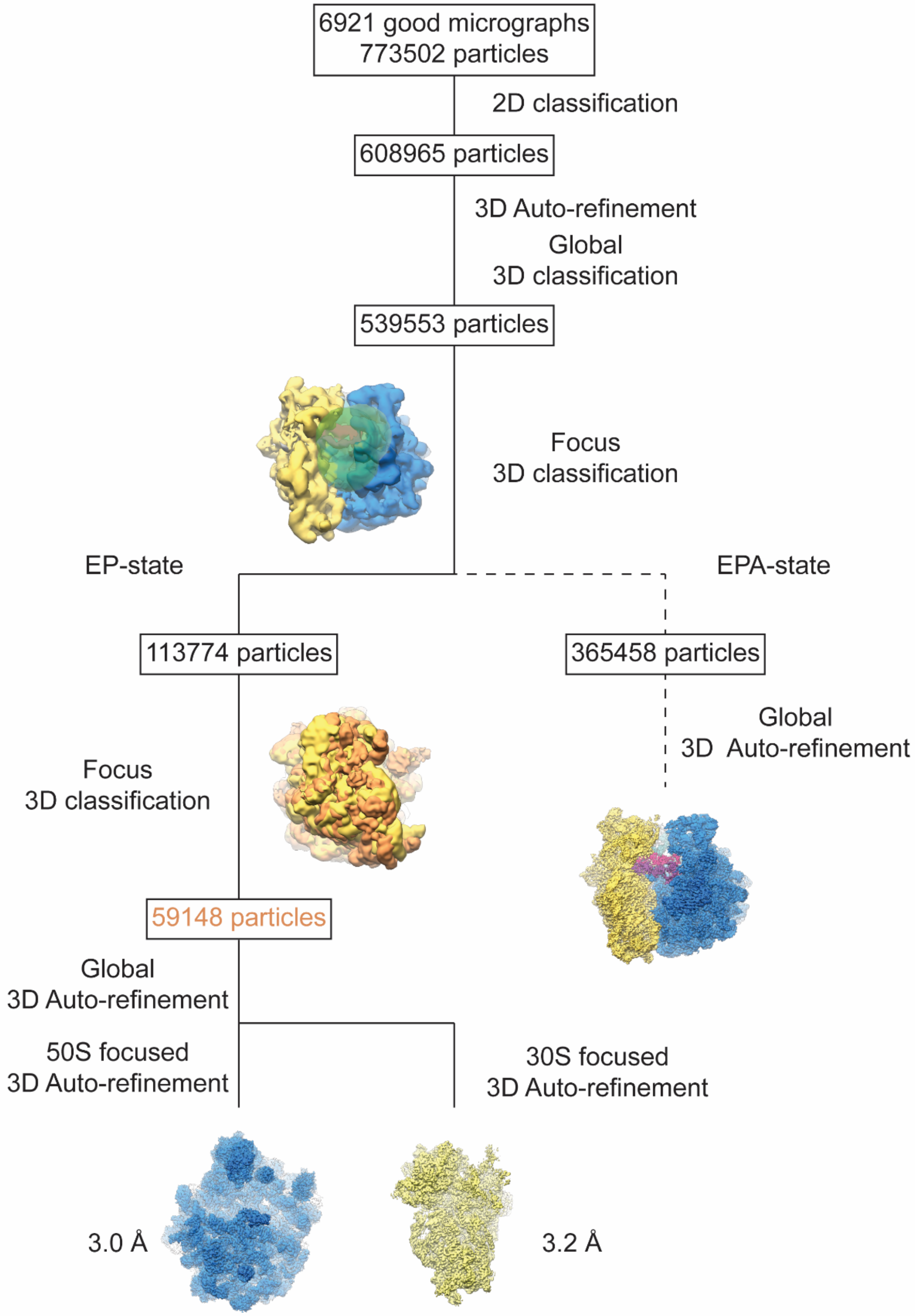
6921 good micrographs
773502 particles
2D classification
608965 particles
3D Auto-refinement
Global
3D classification
539553 particles
Focus
3D classification
EP-state
EPA-state
113774 particles
365458 particles
Global
3D Auto-refinement
Focus
3D classification
59148 particles
Global
3D Auto-refinement
50S focused
3D Auto-refinement
30S focused
3D Auto-refinement
3.0 Å
3.2 Å

Supplementary Figure S3: Classification and refinement scheme for 70S ribosomes in complex with cadazolid, mRNA, tRNA(fMet)$_i$, and Phe-tRNA(Phe)

From 6921 selected micrographs, 773502 particles were picked and classified. Out of these, 608965 good particles were selected from the quality of their class averages and refined against an empty 70S density. A 3D classification was then performed using a mask around the intersubunit space. The subset of particles containing two tRNAs (EP-state) was refined (to align the particles) and 3D classified for the ratchet state of the 70S ribosome. From this, 59148 particles were selected for further refinement, first globally, then with a mask either around the 50S or the 30S subunit.

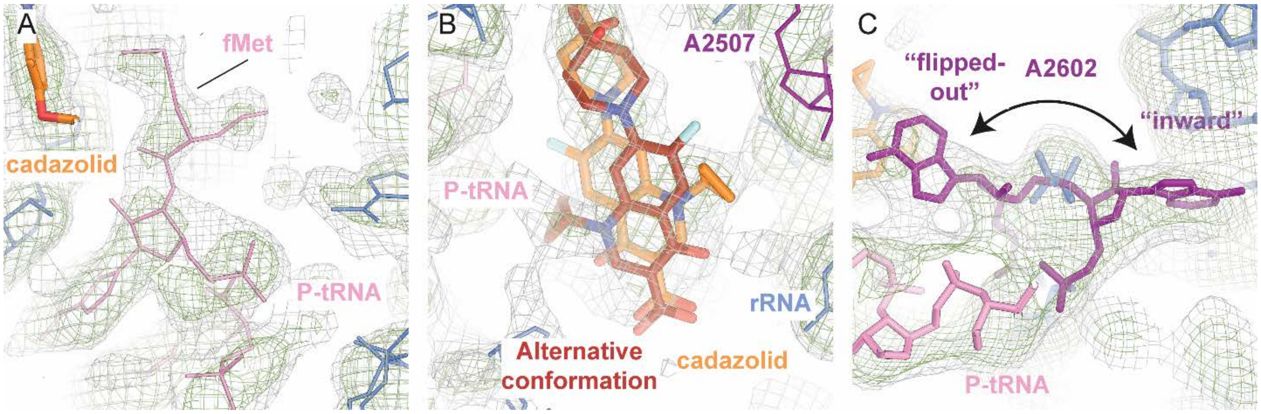

Supplementary Figure S4: Close-up views of cadazolid, P-site tRNA and A2602 in the cryo-EM density. The maps filtered at the overall resolution of 3.0 Å (panels A and B) and the map filtered at 3.5 Å (panel C) are shown at two different contour levels (grey and green).

(a) Density of the CCA-end of the tRNA loaded with fMet (pink).
(b) Two plausible conformations for the fluoroquinolone moiety of cadazolid (orange and red), in which the cyclopropyl group points either towards the P-site tRNA or A2507.
(c) Two visible conformations for the rRNA nucleotide A2602. The "inward" conformation points towards C1965 while the "flipped-out" conformation stacks on top of the fluoroquinolone moiety of CDZ.

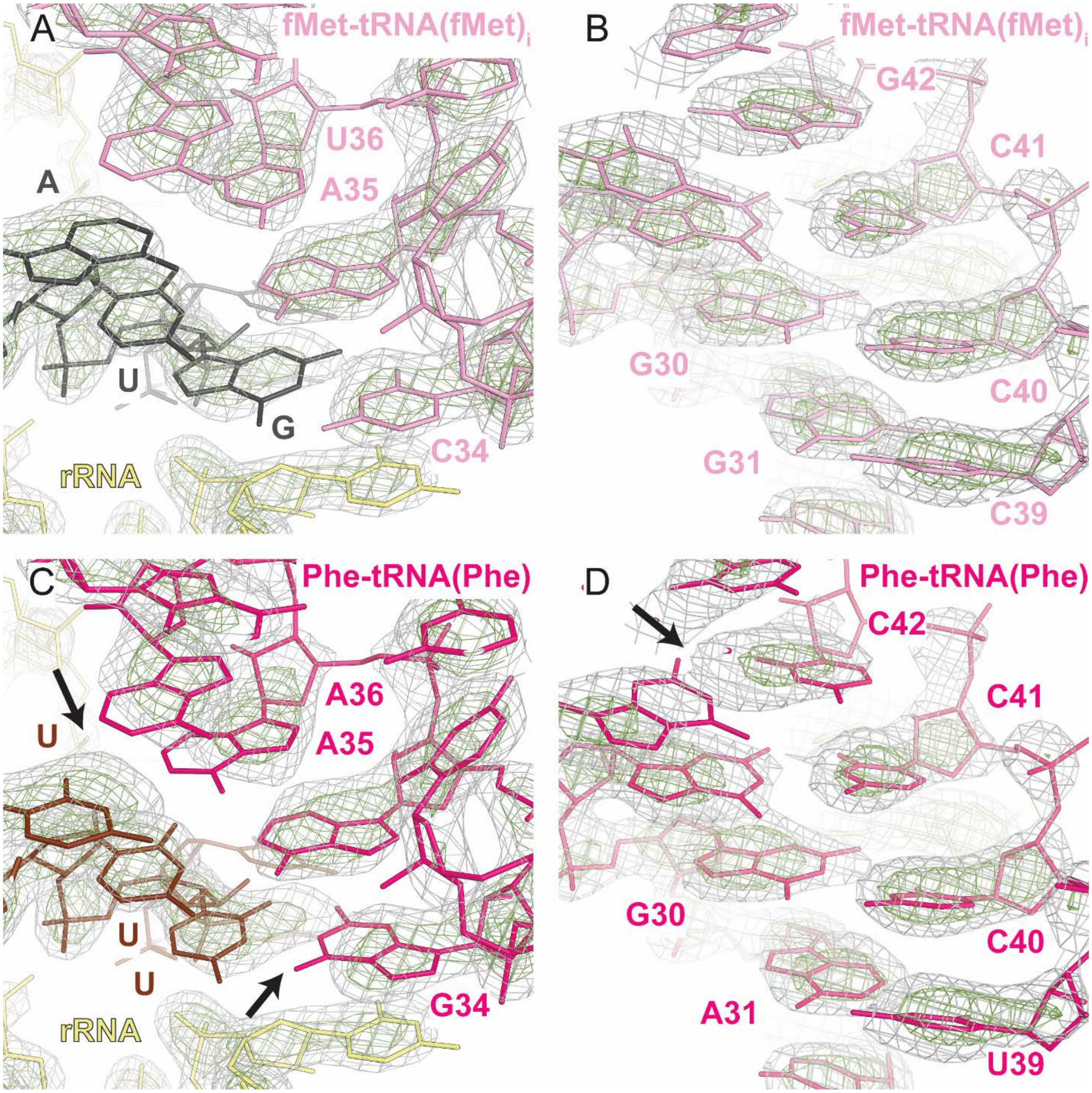

Supplementary Figure S5: Close-up views of mRNA and P-site tRNA in the cryo-EM density to confirm their identity. The maps filtered at the overall resolution of 3.3 Å are shown at two different contour levels (grey and green).

(a) Base pairing between the AUG codon of the mRNA (black) and the anticodon of the P-site fMet-tRNA(fMet)$_i$ (pink).
(b) Further up in the anticodon stem loop of the tRNA (pink), the base pairing pattern specific for fMet-tRNA(fMet)$_i$ confirms the nature of the tRNA.
(c) Same view as panel A showing the base pairing between a UUU codon of the mRNA (brown) and the anticodon of a P-site Phe-tRNA(Phe) (dark pink). The arrows highlight the differences to the density.
(d) Same view as panel B showing the anticodon stem loop of a Phe-tRNA(Phe) (dark pink). The arrows highlight the differences to the density.

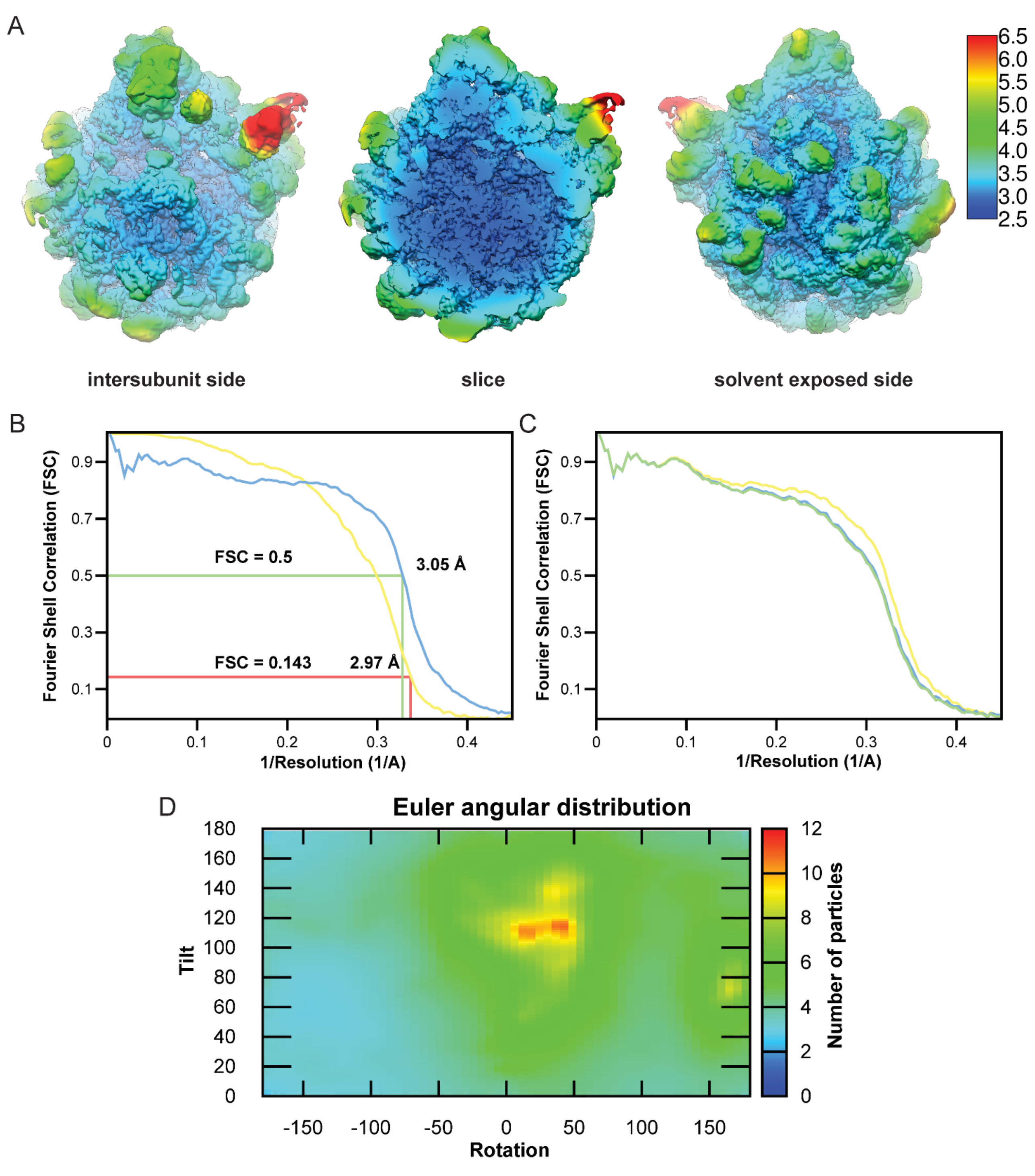

Supplementary Figure S6: Resolution distribution of the reconstruction with a mask around the 50S subunit

(a) Heat map of the local resolution shown for two orientations and a cut through. The local resolution varies between 2.5 Å in the centre containing the PTC to 4-5 Å in the periphery.

Particularly prominent is the L7/L12 stalk, which is flexible and shows a local resolution higher than 6 Å.

(b) Fourier shell correlation (FSC) curve for the cryo-EM map (yellow) and the one calculated from the model versus the map (blue). The EM map resolution was estimated to be 2.97 Å using the gold-standard criterion (FSC=0.143) and coincides perfectly with the value at FSC=0.5 for the model versus map.

(c) FSC curve for the model refined in the density calculated from half of the particles. The FSC was calculated using this model versus the map calculated with all the particles (yellow), against the map from the same half set (green) and against the map from the other half set (blue). The absence of overfitting during the refinement is indicated by the excellent consensus between the three FSC curves.

(d) Heatmap plot of the angular distribution of the particles in the final reconstruction, showing little preferential orientation.
